# Influence of Elevated Potassium Fertilization on Structural and Functional Properties of Sweet Potato Root Tuber Starch

**DOI:** 10.3390/foods13233890

**Published:** 2024-12-02

**Authors:** Ke Guo, Shuai Liu, Long Zhang, Qian Zhang, Yang Yu, Peiyong Ma, Zhaodong Jia, Cunxu Wei, Xiaofeng Bian

**Affiliations:** 1 Institute of Food Crops, Jiangsu Academy of Agricultural Sciences, Nanjing 210014, China; 18115657147@163.com (K.G.); 18761698534@163.com (S.L.); zhangqian508@163.com (Q.Z.); fairyyu88@126.com (Y.Y.); andympy@163.com (P.M.); jzdgood162@126.com (Z.J.); 2 Key Laboratory of Crop Genetics and Physiology of Jiangsu Province/Jiangsu Key Laboratory of Crop Genomics and Molecular Breeding, Yangzhou University, Yangzhou 225009, China; zhanglong@yzu.edu.cn; 3 Co-Innovation Center for Modern Production Technology of Grain Crops of Jiangsu Province, Joint International Research Laboratory of Agriculture & Agri-Product Safety of the Ministry of Education, Yangzhou University, Yangzhou 225009, China

**Keywords:** sweet potato, potassium fertilizer, starch, structural properties, functional properties

## Abstract

Nine sweet potato varieties with different flesh colors were cultivated under uniform environmental conditions with potassium (K) fertilizer treatments at levels of 0, 22.5, and 45 kg/ha. The structural and functional properties of the starches were subsequently analyzed. The soluble sugar content in the dry root tuber increased, with higher K levels in most varieties. Amylose content decreased in Sushu16 but increased in Ningzishu1, with no significant differences observed in other varieties across different K levels. Elevated K levels had no effect on starch protein content, crystalline type, or gelatinization enthalpy. The impact of K fertilizer on starch thermal and pasting properties varied among the varieties. PLSR and PLS-DA analyses revealed that genotype background was the primary factor influencing starch properties. This research will provide a reference for the improvement of sweet potato production quality and efficiency and a scientific basis for the cultivation and utilization of sweet potato root tubers.

## 1. Introduction

Sweet potato (*Ipomoea batatas* (L.) Lam) is an important food crop in many tropical and subtropical regions, and its high yield and resistance to barrenness make it a key factor in food security and industrial applications, particularly in areas with limited resources and harsh environmental conditions [1]. Fertilizer is one of the most important inputs for increasing the sweet potato yield, and potassium (K) is considered the second largest fertilizer needed for plant growth after nitrogen [2,3]. The role of potassium is to speed up the operation of photosynthetic products, regulate the concentration of photosynthetic products, enhance photosynthetic capacity at any time, directly enlarge root tubers, and increase yields [4]. Compared with other commercial crops, sweet potato needs more K content for optimum production [2,5].

Starch, as the main component of sweet potato root tubers, accounts for 50–80% of dry weight and is widely applied in food and non-food industries [6]. Its structural and functional properties can affect the quality and application of sweet potato root tubers. For instance, a reduction in the particle size of sweet potato results in a more compact and stable noodle structure. Furthermore, various factors such as crystalline type, lamellar structure, granule size distribution, and the ratio of amylose to amylopectin significantly influence the digestion properties of sweet potato starch [7,8]. Fertilization, as a key strategy for starch customization, can provide clean-label foods and ingredients at a large scale and low cost [9]. The effects of K fertilization treatment on starch properties have also been reported in rice [10], wheat [11], and potato [12]. The effects of K fertilization treatment on starch properties showed that higher K contents can reduce amylose content in rice [10] and wheat [11]. Studies of potato starch under K fertilization treatment (135, 270, and 405 kg/ha) showed that increasing K fertilization levels decreased amylose content, phosphorus, and granule size but enhanced relative crystallinity [12]. Previous research on the effect of K fertilization on sweet potato primarily focused on its tuber yield, starch content, chlorophyll content, net photosynthetic rate, endogenous hormones, biosynthesis-related enzyme activity of sucrose, and starch and lignin pathways [2,4,5,13,14,15,16,17]. For instance, two sweet potato varieties “Ningzishu1 (K susceptible)” and “Xushu32 (K tolerant)” were treated according to six K_2_O rates: 0, 75, 150, 225, 300, and 375 kg/ha. The results indicated that K fertilization enhanced biomass accumulation and photosynthetic capacity. The activities of sucrose synthase (SuSy) and starch synthase (SSS) all increased under K treatment, which could explain the observed increase in biomass content and yield [14]. Furthermore, transcriptomic analysis revealed that “Xushu32” had a higher number of differentially expressed genes (DEGs) compared to “Ningzishu1”. These DEGs were involved in various metabolic pathways such as photosynthesis, carbohydrate metabolism, and ion transport, which may have contributed to the greater potassium tolerance of “Xushu32” [17]. However, there are few studies that have investigated the effects of K fertilizer on the structural and functional properties of sweet potato starch. Three edible sweet potato varieties were treated with and without potassium application, and the results showed that the peak viscosity and breakdown viscosity significantly decreased in these three varieties without potassium application [18]. Sweet potato, a hexaploid crop with a complex genetic background, presents challenges in genetic and functional studies [19]. Its genotype background can significantly affect the structural and functional properties of starch [6]. Therefore, it is particularly important to compare the effects of K fertilizer treatments on starch structural and functional properties for different genotype backgrounds. We hypothesized that genotype background plays an important role in the effect of K fertilizer treatment on starch properties.

In the present study, nine sweet potato varieties—three with white flesh, three with yellow flesh, and three with purple flesh—with different genotype backgrounds were cultivated based on three potassium levels to assess the effects of K fertilizer on various starch properties, including starch granule size, amylose content, crystalline structure, thermal properties, and pasting properties. The purpose of this study was to elucidate the impact of K fertilizer on sweet potato starch structural and functional properties, thereby establishing a scientific rationale for the post-harvest utilization of sweet potato root tubers.

## 2. Materials and Methods

### 2.1. Plant Materials

In this study, three sweet potato varieties with white flesh (Sushu 24 (W1), Sushu 28 (W2), and Sushu 29 (W3)), three sweet potato varieties with yellow flesh (Sushu 14 (Y1), Sushu 16 (Y2), and Sushu 25 (Y3)), and three sweet potato varieties with purple flesh (Ningzishu 1 (P1), Ningzishu 2 (P2), and Ningzishu 4 (P3)) were selected and planted at the field of Jiangsu Academy of Agricultural Sciences in Luhe District, Nanjing, China (N32°48′72″, E118°64′09″), in 2017. The local altitude is 8.9 m, with an annual solar irradiation range of 2087.3 to 2296.1 h and an atmospheric pressure of 100.4 kPa. The sweet potato seedlings were planted on May 28 and harvested on October 15. During the growth stage, temperature and rainfall information were recorded and are presented in Figure S1. The soil was a typical magan soil, containing 15.2 g/kg of organic matter, 97.6 mg/kg of alkaline nitrogen, 12.4 mg/kg of available phosphorus, and 61.5 mg/kg of quick-acting potassium, with a pH of 5.96. Prior to planting, nitrogen (90 kg/ha of a 46% N fertilizer) and phosphorus (90 kg/ha of a 12% P_2_O_5_ fertilizer) were applied as basal fertilizers. The experiment was conducted in a randomized plot design with three replications, each plot covering an area of 14.4 m^2^ and featuring four rows with a spacing of 0.9 m. Individual seedlings were planted, and protection rows were established around the experimental plot. The soil was treated with three levels of potassium (50% K_2_O): 0 kg/ha (K0), 22.5 kg/ha (K1), and 45 kg/ha (K2) (Table 1). After harvest, the fresh tubers were immediately utilized as experimental materials.

### 2.2. Soluble Sugar and Starch Content of Dry Root Tubers

Fresh root tubers were washed, peeled, and sliced into thin pieces. These pieces were freeze-dried at −70 °C using a freeze-dryer (FD-1A-50, Beijing Boyikang Co., Ltd., Beijing, China). The dried samples were ground into a fine powder and then sieved through a 100-mesh sieve for subsequent use. Soluble sugars were extracted using 80% ethanol. Following the extraction of soluble sugars, the residual starch was subjected to acid hydrolysis with 3 mL of 9.2 M perchloric acid for 15 min. The mixture was then centrifuged at 5000 rpm for 5 min, and the supernatant was collected for further testing. The sediment was further treated with 6 mL of 4.6 M perchloric acid for continued acid hydrolysis. After centrifugation, the supernatant was collected, and this process was repeated three times. The soluble sugar and starch contents in the sample powders were determined using the method of anthrone–H_2_SO_4_ colorimetry following a previously reported method [6].

### 2.3. Starch Isolation

Starch isolation was performed by following a previous protocol [20]. Fresh sweet potato root tubers were harvested and thoroughly washed to remove any dirt, debris, or microbial contaminants. The clean root tubers were then blended in H_2_O and filtered through 100 (150 μm)-, 200 (75 μm)-, and 300 (48 μm)-mesh sieves. The suspension was precipitated after being rested for over 3 h. The sedimented starch was washed multiple times with water to remove any remaining impurities. The purified starch was thoroughly suspended in anhydrous ethanol to eliminate water, followed by centrifugation at 5000× *g* for 5 min. The supernatant was discarded, and this process was repeated three times to ensure complete dehydration. Subsequently, the starch was dried in an oven at 40 °C for 2 d. The dried starch was passed through a 100-mesh sieve, stored in airtight containers at room temperature, and measured immediately.

### 2.4. Starch Composition Determination

The protein content of the starch was analyzed using a method previously established by our group [6]. Briefly, the protein content of starch was calculated by multiplying the nitrogen content by 6.25, while the nitrogen content was determined using a CHN element analyzer (Vario EL cube, Elementar Analysensysteme GmbH, Frankfurt, Germany). The apparent amylose content was determined using the iodine colorimetric method [21]. Next, 10 mg of starch was dissolved in 5 mL urea–dimethyl sulfoxide (UDMSO) solution at 95 °C for 1 h before being colored with an I_2_-KI solution. The absorbance of the resulting solution was measured at 620 nm using a spectrophotometer (Ultrospec 6300 pro, Amersham Biosciences, Piscataway, NJ, USA). The apparent amylose content was estimated by comparing the absorbance of the sample to that of a standard curve constructed with various ratios of potato amylose (A0512, St. Louis, MO, USA, Sigma) and maize amylopectin (A0456, Tokyo, Japan, TCI) standards.

### 2.5. Granule Size Distribution Analysis of Starch

The granule size distribution was measured using a laser particle size analyzer (Mastersizer 2000, Malvern, Worcestershire, UK) [22]. For this, 100 mg of starch was mixed with 5 mL of water to evenly disperse the starch, avoiding agglomeration. During the measurement process, the background was measured to eliminate the influence of impurities in the background. Subsequently, the well-dispersed starch sample was added into the sample cell until the opacity was between 10% and 11%, at which point, the measurement could begin. Three replicate results of surface- and volume-weighted diameters were automatically output by the instrument.

### 2.6. X-Ray Diffraction

The starch crystalline structure was measured using an X-ray diffractometer (D8, Bruker, Karlsruhe, Germany) [23]. Before testing, the starch and saturated NaCl aqueous solution were placed in a sealed container and equilibrated to a humidity of 75% for 2 weeks. The samples were scanned at a diffraction angle of 2θ from 3° to 40°, with a scanning step of 0.02°, a scanning time of 20 min, a voltage of 40 kV, and a current of 40 mA. The relative crystallinity (RC) was determined by utilizing Photoshop CC 2019 software to measure the areas of both the crystalline (a) and amorphous regions (b), followed by the application of the formula RC (%) = a/(a + b) × 100%.

### 2.7. Fourier Transform Infrared (FTIR)

The starch short-range ordered structure was determined using a Varian 7000 Fourier transform infrared (FTIR) spectrometer (7000, Varian, Santa Clara, CA, USA) [24]. The samples were scanned 64 times from 4000 to 800 cm^−1^ with a resolution of 2 cm^−1^, using water as a blank to subtract the background. The spectral baseline was selected as 1200–800 cm^−1^, with a half-peak width of 19 cm^−1^ and an enhancement factor of 1.9. The spectrum was deconvoluted using OMNIC software (Version 8.2.0.387).

### 2.8. Thermal Properties

The starch thermal properties were analyzed using a differential scanning calorimeter (DSC, 200-F3, NETZSCH, Selb, Germany) following a previous report [20]. First, 5 mg of starch was mixed with 15 mL of water to achieve a starch-to-water ratio of 1:3 (*w*/*v*) and placed in a DSC pan. The DSC pan was then hermetically sealed to prevent moisture loss during the measurement. The procedure involved heating the sample from 20 °C to 110 °C at a rate of 10 °C/min.

### 2.9. Pasting Properties

Starch (2.5 g) was mixed with 25 mL of distilled water in an aluminum canister, and the pasting properties were analyzed using a Rapid Viscosity Analyzer (RVA, Perten, Warriewood, NSW, Australia) following a previous report [20]. Briefly, to prepare the sample, 2.5 g of starch was accurately weighed out and mixed with 25 mL of deionized water in an RVA canister, sprinkling the starch carefully to prevent clumping. The prepared canister was securely fastened onto the RVA unit. The specific test profile included holding at 50 °C for 1 min, heating to 95 °C at a rate of 12 °C per minute, maintaining at 95 °C for 2.5 min, cooling back to 50 °C at a rate of 12 °C per minute, and, finally, holding at 50 °C for an additional 1.4 min.

### 2.10. Statistical Analysis

This study assessed sample differences through the application of Tukey’s test using SPSS 19.0. Partial least squares regression (PLSR) of starch structural and functional property parameters and partial least squares discriminant analysis (PLS-DA) of starches were conducted using SIMCA 14.0 software.

## 3. Results and Discussion

### 3.1. Soluble Sugar and Starch Content of Dry Root Tubers

The analysis of nine sweet potato varieties with three K fertilization levels revealed significant variations in both soluble sugar and starch content (Table 2). For the soluble sugar content, an overall increasing trend with higher K levels was observed in several varieties. Specifically, most of the varieties, such as W1, W2, Y1, Y3, P2, and P3, showed a notable increase in soluble sugar content with increased K fertilization. For example, the soluble sugar content of Y1 increased from 31.5% at K0 to 33.7% at K1 and Y3 increased from 28.9% at K1 to 33.8% at K2. Conversely, W3 showed no significant change under different levels of K treatment. Interestingly, the soluble sugar content of P1 rose from 22.4% at K0 to 24.8% at K1, followed by a slight decrease to 17.9% at K2. This may have been due to different genotype background varieties having different responses to K treatment. The response of starch content to K fertilization was more variable. While some varieties, such as W1 and Y2, showed an initial decrease in starch content with increased potassium levels, other varieties, such as W2, P1, and P3, exhibited an increase. For instance, the starch content of W1 decreased from 67.5% at K0 to 65.3% at K1 and remained relatively stable at 65.4% at K2. In contrast, W2 and P3 increased their starch content from 61.5% at K0 to 65.4% at K2 and from 53.0% at K0 to 55.6% at K2, respectively. On the other hand, the starch content of P2 increased from 55.2% at K0 to 56.1% at K2, showing a more consistent response to potassium fertilization. According to the present results, varieties such as Y1 and Y3, which showed significant increases in soluble sugar content, might be more suitable for applications requiring higher sweetness. In contrast, varieties such as W2 and P3, which demonstrated an increase in starch content with higher potassium levels, could be more appropriate for industrial uses where a higher starch yield is desired.

The response of sweet potato varieties to potassium fertilization in terms of soluble sugar and starch content is complex and variety-specific. Previous research indicated that an adequate supply of K increased the photoassimilate transportation rate from leaves to roots and enhanced nutrient use efficiency due to the influence of photosynthesis in apples [25]. The increase in soluble sugar content with higher potassium levels in most varieties suggested that potassium plays a role in enhancing photosynthetic capacity, thereby increasing carbohydrate metabolism and potentially improving the sweetness and quality of the tubers. Another possible explanation is that K treatment can suppress the synthesis of other components in sweet potato tubes, such as the lignin biosynthesis pathway [15], leading to more active sugar synthesis. However, the variable response in starch content indicates that other factors, possibly genetic or environmental, influence starch biosynthesis and accumulation. In the present research, the application of K increased soluble sugar content for most varieties; however, for starch content, the response was shown to be genotype-dependent. Overall, the differential responses underscore the importance of tailored fertilization strategies to optimize the desirable traits in sweet potato varieties for specific end uses.

### 3.2. Protein and Apparent Amylose Content of Starch

Protein and amylose are the main components of starch. The protein content (PC) and apparent amylose content (AAC) across different sweet potato varieties under varying K fertilization treatments are shown in Table 3. Protein content remained relatively stable across all treatments, with only slight variations observed, except P3, which showed a marked increase from 0.031 mg/g to 0.044 mg/g. Notably, Y1 and P1 maintained higher protein contents across all K levels, with Y1 consistently around 0.050 mg/g and P1 ranging from 0.047 to 0.050 mg/g. Typically, the protein content in starch depends on the species and variety of starch, and it is generally very low, particularly in root or tuber starch [26]. Additionally, during the starch isolation process, part of the surface proteins can be removed through washing procedures, further contributing to the low protein content in starch [27]. These results suggested that potassium fertilization does not markedly influence the protein content in sweet potato varieties, maintaining relative stability across different levels, which indicated that protein synthesis in sweet potato tubers was relatively unaffected by K variations. This could imply that the pathways regulating protein synthesis were not significantly influenced by potassium, or that the existing levels of potassium in the soil were sufficient to meet the protein synthesis needs of the sweet potato varieties under study. This stability is beneficial for maintaining the nutritional quality of sweet potatoes as consistent protein content is crucial for both human consumption and livestock feed.

In terms of apparent amylose content, the majority of varieties showed no significant response to K treatment (Table 3). This includes all three white-fleshed varieties (W1, W2, W3), two yellow-fleshed varieties (Y1, Y3), and two purple-fleshed varieties (P2, P3). However, slight fluctuations were observed in Y2 and P1 with varying K treatments. Y2 presented decreased AAC from 28.4% at K0 to 26.0% at K1, which then slightly increased to 27.2% at K2. Different from Y2, P1 presented an increasing tendency from 22.8% at K0 to 24.6% at K2. In the previous study, two sweet potato varieties, “Xushu32” and “Ningzishu1”, were subjected to varying rates of K_2_O (0, 75, 150, 225, 300, and 375 kg K_2_O/ha) during the years 2017 and 2018. The findings revealed a consistent decrease in amylose content with increasing potassium application rates across both years [14]. In another study, two potato varieties, “Zhongshu5” and “Atlantic”, were treated with three K application rates (135, 270, and 405 kg/ha), and the results indicated that the amylose content was decreased after K treatment. In the present research, most of the varieties showed no effect after K treatment; only Y2 showed a decreasing tendency. This disagreement is mainly because the genotype background is an important factor that can affect starch properties [6]. The increase in amylose content in P1 indicated that higher potassium levels may enhance the synthesis or accumulation of amylose. This could be due to the role of K in enzyme activation and metabolic processes that favor starch biosynthesis.

Overall, the variety-specific responses highlight the genetic diversity among sweet potato cultivars and their unique metabolic pathways. For example, while most cultivars exhibited no significant changes in PC and AAC in response to K fertilization, the notable reduction in amylose content with increasing potassium levels in Y2 suggested a differential sensitivity to potassium availability. This phenomenon implied that this variety may have a lower threshold for optimal potassium levels, beyond which, amylose synthesis is inhibited or redirected. These findings emphasize the necessity for customized fertilization strategies tailored to the specific requirements and responses of individual sweet potato varieties. Such precision in agricultural practices contrasts with a generalized fertilization approach, advocating instead for strategies that maximize both yield and nutritional quality, thereby enhancing the overall agricultural and economic viability of sweet potatoes.

### 3.3. Granule Size Distribution of Starch

The granule size distribution of nine sweet potato varieties was analyzed using a laser diffraction granule size analyzer. The results, which are shown in Table 3, revealed significant variability among varieties and treatments. The different-colored sweet potato starches showed different granule sizes; W2 showed the highest D [4,3] and Y3 had the lowest D [4,3] without K treatment. Generally, higher potassium levels (K1 and K2) tended to increase starch granule size compared to the control (K0) across most varieties. For instance, W2, Y3, and P3 showed first increases and then slight decreases in terms of D [4,3], while P2 showed increases among the different K treatments. W1, W3, and P1 first showed decreases and then slight increases in terms of D [4,3], and Y2 exhibited smaller starch granules with increasing K application. The notable exception was Y1, where K treatments showed less pronounced effects on granule size distribution. Two potato varieties, “Zhongshu5” and “Atlantic”, were treated with three different K levels (135, 270, and 405 kg/ha), and a higher level of K content (405 kg/ha) significantly enlarged the average diameter of the starch (45.7 μm→43.1 μm and 47.6 μm→44.9 μm) [12]. The observed variability in starch granule size distribution among the sweet potato varieties under different K treatments highlighted the influence of genetic factors. Furthermore, investigation into the underlying biochemical pathways and regulatory mechanisms governing starch development in response to different K levels, such as lignin biosynthesis, as mentioned earlier, could provide deeper insights into the broader effects of K on sweet potato physiology and facilitate the potential trade-offs or synergies in nutrient allocation, further refining fertilization practices.

### 3.4. X-Ray Diffraction of Starch

**Figure 1:**
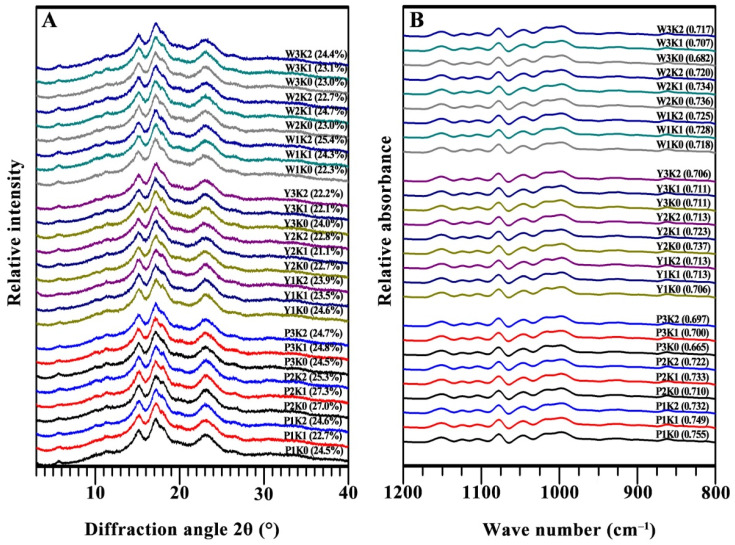
XRD patterns (**A**) and ATR-FTIR spectra (**B**) of starch. Values in parentheses are shown for relative crystallinities (RCs) (**A**) and ordered degrees (ODs) of starch, as calculated by the absorbance ratios of 1047 and 1022 cm^−1^ (**B**).

The crystalline type and relative crystallinity of starches treated under different K levels were detected using X-ray diffraction (see Figure 1A). The X-ray diffraction (XRD) patterns of starches revealed distinct peaks that differentiated A-type, B-type, and C-type starches. Specifically, A-type starch displayed prominent peaks at 2θ of 15°, 17°, 18°, and 23°. In contrast, B-type starch exhibited peaks at 2θ of 15°, 17°, 22°, and 24°, along with a distinctive peak at 5.6°. C-type starch, meanwhile, combined the characteristics of both A-type and B-type starches. Depending on the relative proportions of A-type and B-type crystallinity, C-type starch can be subclassified into C_A_-, C_B_-, and C_C_- types [28]. In the current research, starches exhibited prominent diffraction peaks at 2θ of 15°, 17°, and 23°, accompanied by a weak characteristic peak at 2θ of 5.6° and a shoulder peak at 2θ of 18°. The relatively weaker peak at 5.6° and the stronger peaks at 17° indicated a stronger percentage of A-type starch characteristics, suggesting that their classification was typical C_A_-type starch. The results revealed that K treatment had no effect on starch crystalline type, regardless of whether the K level was higher or lower and regardless of different genotype backgrounds. Similar results have also been reported for sweet potato [29] and potato [12], where fertilizer application did not alter crystalline type. Starch crystalline type was mainly affected by genotype background, amylopectin branch chains, and growth environment, especially growth temperature, which played a more important role than amylopectin branch chains [20,30]. In the present research, all sweet potato varieties were grown in the same place and controlled according to the same agronomy practice, which led to the same crystalline type.

Compared with the crystalline type, the relative crystallinity exhibited diverse variations (Figure 1A). The K treatments induced marginal alterations in relative crystallinity; however, W1 demonstrated a significant increase, whereas Y3 and P2 exhibited substantial decreases. Starch relative crystallinity can be affected by many factors, while granule size is a key factor influencing relative crystallinity. As previous research reported, starch granule size shows a negative correlation with relative crystallinity. Starch relative crystallinity can be influenced by numerous factors, with granule size being a critical determinant. Prior research has demonstrated a negative correlation between starch granule size and relative crystallinity [31]. The present results indicated that the granule size decreased in W1 and increased in Y3 and P3 with rising K levels (Table 3), which likely contributed to the observed variations in relative crystallinity.

### 3.5. ATR-FTIR Spectra of Starch

The short-range ordered structure was analyzed using ATR-FTIR spectroscopy, with the data acquisition covering the spectral range from 4000 to 800 cm^−1^ and a detailed deconvolution performed between 1200 and 800 cm^−1^. The ATR-FTIR spectra revealed characteristic absorption bands associated with various starch molecular vibrations. Generally, peaks in the 3300–3600 cm^−1^ region represented the OH stretching vibrations, those in the 2900–3000 cm^−1^ region corresponded to C-H stretching vibrations, and the bands from 1100 cm^−1^ to 1500 cm^−1^ were indicative of C-O, C-C, and C-O-H stretching modes. Additionally, the region between 900 cm^−1^ and 1100 cm^−1^ was attributed to C-O-H bending and stretching vibrations [32]. The absorbance bands at 1047 cm^−1^ and 1022 cm^−1^ are particularly significant in the context of starch structure. The band at 1047 cm^−1^ is generally associated with the ordered structure of starch, while the band at 1022 cm^−1^ corresponds to the amorphous regions [33]. This distinction is crucial for assessing the ordered degree within starch, as the ratio of these peaks—1047 cm^−1^ to 1022 cm^−1^—serves as a quantitative measure of starch ordered structure [34], which provides a basis for the evaluation of starch structural changes. In the present study, starch samples subjected to varying levels of potassium (K) fertilization exhibited FTIR spectra with similar general features (Figure 1B), indicating that K fertilization did not introduce new functional groups or fundamentally alter the molecular composition of the starch. However, the ratio of the 1047/1022 cm^−1^ peak intensities, which reflects the degree of structural order, varied among different samples (Figure 1B). Notably, a slight increase in this ratio was observed in some samples (W1, W3, Y1, P1, and P2), suggesting a modest enhancement in the ordered structure of starch due to K treatment. Conversely, a slight decrease in the ratio was noted in samples Y2 and P3, indicating a reduction in structural order. Other samples did not show significant changes in the ratio of 1047/1022 cm^−1^. These findings suggested that the effect of K fertilization on the ordered structure of starch was not uniform across different varieties of sweet potato, and the variability in the 1047/1022 cm^−1^ ratio indicated that different sweet potato varieties respond differently to K fertilization, reflecting inherent differences in their starch physiology and crystallinity. Such variations may be attributed to the genetic and physiological differences among the varieties, which influence the assimilation and utilization of potassium.

### 3.6. Thermal Properties of Starch

The starch thermal properties were measured using DSC and are shown in Table 4. The DSC data revealed that potassium fertilization influenced the thermal properties of sweet potato starch, with variations in gelatinization onset temperature (To), gelatinization peak temperature (Tp), gelatinization conclusion temperature (Tc), and gelatinization enthalpy (ΔH). For starches from W2 and P1, an increase in potassium levels significantly elevated the To, while starches from W3 and P2 exhibited a noticeable decrease. Notably, yellow-fleshed sweet potato starches showed no response to potassium treatment. The Tp, a critical parameter representing the temperature at which most starch granules gelatinize, significantly increased with higher potassium levels in W1, Y1, and Y3 but decreased in W2, W3, Y2, and P2. Additionally, P1 exhibited first a decreasing pattern and then an increasing pattern with rising potassium levels, whereas P3 showed first an increasing trend and then a decreasing trend. Gelatinization enthalpy (ΔH), which denotes the energy required for starch gelatinization, showed no significant variation except for an increase in P2 under different potassium treatment levels. Previous research on the effect of K fertilization on potato starch showed that a higher K level could reduce To, Tp, and Tc but increased ΔH [12]. The present results highlighted that the effect of K fertilizer treatments on sweet potato starch thermal properties depends primarily on the genotype background.

**Figure 2:**
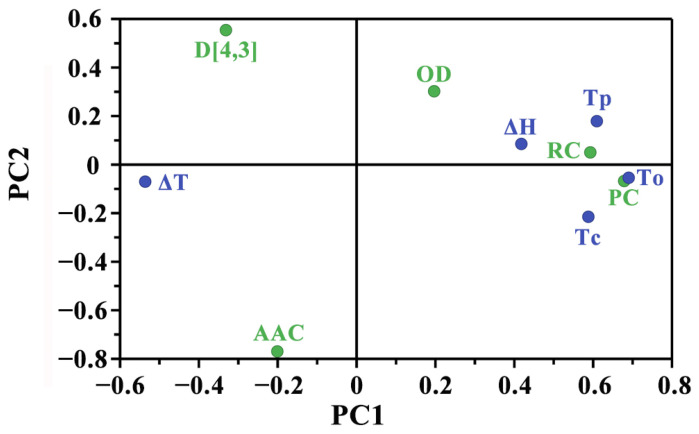
Results of PLSR analysis based on the structural and thermal parameters of starches. Green and blue colors represent the structural parameters and thermal parameters, respectively.

Partial least squares regression (PLSR) is a statistical technique that models the relationship between a set of predictor variables and one or more response variables by extracting latent variables that maximize the covariance between them. The results of the PLSR analysis of starch are shown in Figure 2. The principal components 1 and 2 contributed to 28.4% and 38.0% of the variance, respectively. The PLSR loading plot showed that the relative crystallinity (RC), ordered degree (OD), and protein content (PC) had a significant positive correlation with starch thermal properties including To, Tp, Tc, and ΔH. The apparent amylose content (AAC) showed a negative correlation with starch thermal properties. Apart from this, the granule size (D [4,3]) had a negative correlation with To, Tp, Tc, and ΔH. Previous research also reported that D [4,3] was negatively correlated with starch thermal properties, while the relative crystallinity was positively correlated with thermal properties [27]. In the present research, different responses to K fertilization treatment may have been caused by different granule sizes, protein contents, apparent amylose contents, and relative crystallinities in different varieties.

### 3.7. Pasting Properties of Starch

The effect of K fertilizer treatments on the pasting properties of sweet potato starch was evaluated using a rapid viscosity analyzer (RVA), and the results are summarized in Table 5. The peak viscosity (PV) initially increased and then decreased in W1 and P1, while, in W3, it first decreased and then increased with rising K levels. In contrast, W2 and Y3 exhibited a significant decrease in PV values, whereas P3 showed an increase as K levels increased. Notably, K fertilizer application had no effect on PV in the Y1 and P2 varieties. Hot viscosity (HV) demonstrated a significant increase in W3 and Y1 but a decrease in W1 and P3 with K fertilizer application. In W2, P1, and P2, K fertilizer treatment did not affect HV values. Breakdown viscosity (BV) represents starch pasting resistance to heat. A lower breakdown viscosity value means a greater ability to withstand heat [35]. Generally, an increase in W2, Y2, and P2 was observed, while Y1 and Y3 exhibited no response to K fertilizer treatment. Final viscosity (FV) represents the stability of a paste and its ability to form a gel or maintain its structure after cooling [36]. For most varieties, including W2, Y1, P1, and P2, K fertilizer had no effect on FV values. However, in W3 and Y2, K fertilizer application led to an increase in FV, while, in P3, it resulted in a decrease. Setback viscosity (SV), measured as the difference between the final viscosity (FV) and the hot viscosity (HV), is a crucial indicator of the tendency of starch to experience retrogradation [6]. K fertilizer treatments generally had no significant effect on SV values across most varieties, with the exception of P3, where the SV increased from 801 mPa s to 897 mPa s.

**Figure 3:**
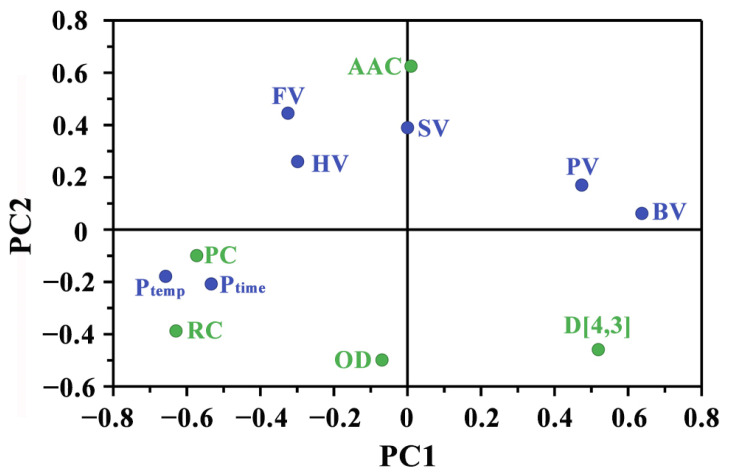
Results of PLSR analysis based on the structural and pasting parameters of starches. Green and blue colors represent the structural parameters and pasting parameters, respectively.

In order to further elucidate the mechanisms underlying the influence of K fertilizer on starch pasting properties, a PLSR analysis incorporating structural and pasting parameters of starches was conducted, as shown in Figure 3. Principal components 1 and 2 contributed to 29.7% and 37.2% of the variance, respectively. This analysis highlighted the complex interrelationships between starch pasting properties and structural characteristics, providing valuable insights into their underlying mechanisms. The FV, HV, and SV aligned more closely with apparent amylose content (AAC), implying that a higher amylose content enhances paste stability and gel formation. Moreover, the relative crystallinity (RC) and protein content (PC) showed a positive correlation with peak temperature (P_temp_) and peak time (P_time_) while demonstrating a negative correlation with PV and BV. This suggested that higher protein content and crystallinity may inhibit starch swelling and gelatinization. Overall, the application of potassium fertilizer exerted a notable influence on the pasting properties of sweet potato starch. The observed changes in viscosity parameters underscored the potential of K fertilizer to modify starch functionality, which could be strategically utilized to optimize starch-based product formulations.

### 3.8. Multivariate PLS-DA of Starches

**Figure 4:**
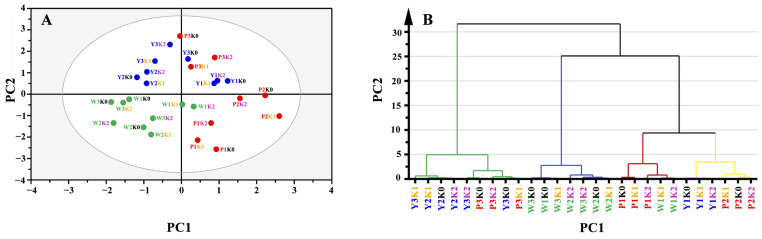
PLS-DA (**A**) and hierarchical cluster analysis diagram (**B**) based on the structural, thermal, and pasting parameters of starches.

The PLS-DA analysis effectively discriminated between sweet potato varieties based on their starch thermal and pasting properties and structural characteristics, emphasizing the impact of K fertilizer treatments (Figure 4A). Principal components 1 and 2 accounted for 28.6% and 38.4% of the variance, respectively. Different colors were used to distinguish between sweet potato varieties—white-fleshed (green), yellow-fleshed (blue), and purple-fleshed (red)—and K fertilization levels—K0 (black), K1 (yellow), and K2 (violet). The distance between any two starches reflected their degree of similarity or difference. Yellow-fleshed varieties, except for Y1, were distinctly grouped together, indicating that these varieties exhibited similar starch properties as influenced by K fertilizer treatments. However, the yellow-fleshed variety Y1 was positioned close to P2 and P3, suggesting that its starch properties were more similar to those of the purple-fleshed sweet potatoes than to other yellow-fleshed varieties. Interestingly, the purple-fleshed sweet potato variety P1 showed similar starch properties to white-fleshed sweet potato varieties W1 and W2. K fertilizer treatment had a minimal effect on W2, Y1, Y2, and P2, as evidenced by the close proximity of the K0, K1, and K2 treatments within each variety, indicating that these varieties could maintain relatively consistent starch characteristics regardless of treatment. However, the white-fleshed varieties W1 and W3, the yellow-fleshed variety Y3, and the purple-fleshed varieties P1 and P3 showed a more scattered distribution of K treatments, indicating a significant modification of starch properties in response to K fertilizer.

To further elucidate the effects of K fertilizer treatment on various varieties, a hierarchical cluster analysis was conducted, and the results are presented in Figure 4B. Principal components 1 and 2 contributed to 49.4% and 25.7% of the variance, respectively. When combined with the PLS-DA results (Figure 4A), the hierarchical cluster analysis revealed distinct clusters among the sweet potato varieties, underscoring significant differences in starch properties in response to varying levels of K fertilizer. The purple-fleshed varieties P1, P2, and P3 with different K fertilizer treatments were primarily grouped together, showing minimal variation across K treatments, while the white and yellow-fleshed varieties were distributed into separate clusters with noticeable distinctions between the different K treatment levels, particularly for W1, W3, and Y3. Overall, this analysis demonstrated that K fertilizer can differentially influence starch functionality depending on the sweet potato varieties, offering opportunities to tailor starch properties for specific end uses.

## 4. Conclusions

In conclusion, the present study investigated the impact of varying levels of potassium (K) fertilizer (K0, K1, and K2) on the starch structural and functional properties of three white-fleshed, three yellow-fleshed, and three purple-fleshed sweet potato varieties. The results demonstrated that while K fertilizer significantly enhanced the soluble sugar content across most varieties, it had minimal effects on starch protein content, apparent amylose content, crystalline type, and gelatinization enthalpy. The observed variations in granule size, relative crystallinity, ordered degree, thermal properties, and pasting properties can be attributed to the unique varietal characteristics and genotypic differences among the sweet potato varieties. These findings suggest that the responsiveness of sweet potato starch properties to K fertilizer treatment is variety-specific and genotype-dependent. Future research on the effects of K fertilizer on sweet potato starch characteristics could better integrate modern agricultural technology and precision nutrition management strategies, thereby enhancing the nutritional and economic value of sweet potatoes and contributing to the sustainable development of agriculture.

## Figures and Tables

**Table 1:** Information on sweet potato varieties and potassium (K) treatments used in this study.

Variety	Abbreviation	Flesh Color	Treatment
Sushu 24	W1	White	K0: 0 kg/ha K1: 22.5 kg/ha K2: 45 kg/ha
Sushu 28	W2		
Sushu 29	W3		
Sushu 14	Y1	Yellow	
Sushu 16	Y2		
Sushu 25	Y3		
Ningzishu 1	P1	Purple	
Ningzishu 2	P2		
Ningzishu 4	P3		

**Table 2:** Soluble sugar and starch content of dry root tubers ^a^.

	Soluble Sugar Content (%)	Starch Content (%)
W1K0	14.6 ± 0.4 a	67.5 ± 1.0 b
W1K1	14.9 ± 0.2 ab	65.3 ± 0.4 a
W1K2	16.0 ± 0.8 b	65.4 ± 0.7 a
W2K0	18.0 ± 0.1 ab	61.5 ± 1.3 a
W2K1	17.2 ± 0.7 a	63.8 ± 0.8 ab
W2K2	19.5 ± 0.9 b	65.4 ± 0.5 b
W3K0	13.1 ± 0.9 a	63.0 ± 1.7 b
W3K1	12.6 ± 0.6 a	65.3 ± 0.3 b
W3K2	14.5 ± 0.9 a	59.4 ± 0.7 a
Y1K0	31.5 ± 0.2 a	45.8 ± 1.1 a
Y1K1	33.7 ± 0.6 b	44.9 ± 1.4 a
Y1K2	32.6 ± 0.4 a	44.1 ± 1.0 a
Y2K0	31.0 ± 0.3 b	53.1 ± 0.4 c
Y2K1	30.1 ± 0.4 b	47.0 ± 0.6 a
Y2K2	27.4 ± 0.8 a	49.8 ± 0.7 b
Y3K0	28.9 ± 0.6 a	48.8 ± 0.7 b
Y3K1	28.3 ± 0.4 a	48.0 ± 1.0 b
Y3K2	33.8 ± 0.2 b	42.7 ± 1.3 a
P1K0	22.4 ± 1.0 b	55.9 ± 1.3 ab
P1K1	24.8 ± 1.5 b	54.3 ± 1.0 a
P1K2	17.9 ± 0.4 a	57.1 ± 0.4 b
P2K0	17.6 ± 0.2 a	55.2 ± 1.6 b
P2K1	18.8 ± 0.5 b	49.2 ± 1.1 a
P2K2	19.2 ± 0.3 b	56.1 ± 0.2 b
P3K0	20.7 ± 0.5 a	53.0 ± 0.3 a
P3K1	26.3 ± 1.3 b	52.6 ± 0.7 a
P3K2	22.4 ± 0.3 a	55.6 ± 1.1 b

^a^ Data are means ± standard deviations (*n* = 3). Values in the same column and the same variety with different letters are significantly different (*p* < 0.05).

**Table 3:** Protein contents, amylose contents, and granule sizes of starches ^a^.

	PC (mg/g) ^b^	AAC (%) ^b^	Granule Size (μm)		
			d (0.5) ^b^	D [3,2] ^b^	D [4,3] ^b^
W1K0	0.022 ± 0.004 a	23.9 ± 0.2 a	14.747 ± 0.009 c	7.480 ± 0.003 c	15.217 ± 0.009 c
W1K1	0.028 ± 0.004 a	24.1 ± 0.3 a	14.121 ± 0.002 a	7.139 ± 0.002 a	14.587 ± 0.002 a
W1K2	0.028 ± 0.004 a	24.4 ± 0.6 a	14.584 ± 0.006 b	7.295 ± 0.003 b	15.129 ± 0.005 b
W2K0	0.031 ± 0.000 a	24.0 ± 0.1 a	17.242 ± 0.005 a	8.510 ± 0.002 a	18.088 ± 0.006 a
W2K1	0.025 ± 0.000 a	24.2 ± 0.5 a	18.138 ± 0.005 c	9.042 ± 0.002 b	18.855 ± 0.005 c
W2K2	0.022 ± 0.004 a	23.5 ± 0.1 a	17.325 ± 0.010 b	8.484 ± 0.106 a	18.186 ± 0.011 b
W3K0	0.022 ± 0.004 a	23.5 ± 0.2 a	16.808 ± 0.002 c	8.115 ± 0.001 c	17.722 ± 0.002 c
W3K1	0.019 ± 0.000 a	23.9 ± 0.2 a	15.542 ± 0.009 a	7.668 ± 0.005 a	16.331 ± 0.010 a
W3K2	0.022 ± 0.004 a	23.4 ± 0.3 a	15.789 ± 0.013 b	7.900 ± 0.007 b	16.663 ± 0.012 b
Y1K0	0.050 ± 0.000 a	25.3 ± 0.5 a	13.712 ± 0.196 b	7.027 ± 0.070 c	14.322 ± 0.275 b
Y1K1	0.047 ± 0.004 a	24.5 ± 1.0 a	13.020 ± 0.247 a	6.665 ± 0.057 a	13.503 ± 0.229 a
Y1K2	0.050 ± 0.000 a	25.6 ± 0.4 a	13.703 ± 0.005 b	6.845 ± 0.003 b	14.438 ± 0.005 b
Y2K0	0.031 ± 0.000 a	28.4 ± 0.2 c	15.529 ± 0.011 c	7.631 ± 0.084 a	16.429 ± 0.009 c
Y2K1	0.044 ± 0.009 a	26.0 ± 0.2 a	15.072 ± 0.003 b	8.137 ± 0.003 c	15.972 ± 0.004 b
Y2K2	0.034 ± 0.004 a	27.2 ± 0.2 b	14.891 ± 0.013 a	7.937 ± 0.009 b	15.790 ± 0.022 a
Y3K0	0.031 ± 0.009 a	26.5 ± 0.9 a	11.701 ± 0.012 a	5.904 ± 0.007 a	12.328 ± 0.020 a
Y3K1	0.031 ± 0.009 a	26.0 ± 0.2 a	12.230 ± 0.007 c	6.121 ± 0.003 b	12.859 ± 0.006 c
Y3K2	0.038 ± 0.009 a	27.3 ± 0.8 a	12.036 ± 0.019 b	6.177 ± 0.008 c	12.417 ± 0.018 b
P1K0	0.047 ± 0.004 a	22.8 ± 0.1 a	17.098 ± 0.006 c	8.466 ± 0.001 c	17.748 ± 0.006 b
P1K1	0.050 ± 0.000 a	22.5 ± 0.7 a	16.632 ± 0.010 a	8.351 ± 0.005 b	17.197 ± 0.012 a
P1K2	0.050 ± 0.009 a	24.6 ± 0.6 b	17.048 ± 0.010 b	8.239 ± 0.007 a	17.924 ± 0.012 c
P2K0	0.044 ± 0.000 a	24.2 ± 0.5 a	12.970 ± 0.011 a	6.562 ± 0.003 b	13.510 ± 0.013 a
P2K1	0.044 ± 0.000 a	23.3 ± 0.6 a	13.040 ± 0.005 b	6.517 ± 0.003 a	13.551 ± 0.007 b
P2K2	0.047 ± 0.004 a	24.6 ± 1.0 a	13.609 ± 0.004 c	6.739 ± 0.002 c	14.406 ± 0.005 c
P3K0	0.031 ± 0.000 a	26.8 ± 0.5 a	11.946 ± 0.002 a	6.169 ± 0.001 a	12.566 ± 0.005 a
P3K1	0.034 ± 0.004 ab	26.5 ± 0.7 a	13.144 ± 0.011 c	6.699 ± 0.004 c	14.113 ± 0.015 c
P3K2	0.044 ± 0.000 b	26.9 ± 1.1 a	12.685 ± 0.010 b	6.478 ± 0.003 b	13.515 ± 0.010 b

^a^ Data are means ± standard deviations (*n* = 3). Values in the same column and the same variety with different letters are significantly different (*p* < 0.05). ^b^ PC, protein content; AAC, apparent amylose content; d(0.5), granule size at which 50% of all the granules by volume were smaller; D [3,2] and D [4,3], the surface- and volume-weighted mean diameter of granules, respectively.

**Table 4:** Thermal parameters of starches ^a^.

	To (°C) ^b^	Tp (°C) ^b^	Tc (°C) ^b^	ΔT (°C) ^b^	ΔH (J/g) ^b^
W1K0	58.0 ± 0.1 b	70.2 ± 0.1 a	84.3 ± 1.2 a	26.3 ± 1.2 a	10.3 ± 0.6 a
W1K1	57.3 ± 0.3 a	70.5 ± 0.6 a	84.7 ± 0.7 a	27.4 ± 0.6 a	11.5 ± 0.5 a
W1K2	58.4 ± 0.3 b	76.5 ± 0.2 b	85.5 ± 0.5 a	27.1 ± 0.8 a	9.9 ± 1.1 a
W2K0	52.1 ± 0.6 a	74.5 ± 0.3 b	83.3 ± 1.0 b	31.3 ± 1.1 b	10.0 ± 0.8 a
W2K1	51.8 ± 0.5 a	75.8 ± 0.1 c	83.6 ± 0.3 b	31.9 ± 0.5 b	9.5 ± 1.0 a
W2K2	53.2 ± 0.3 b	65.3 ± 0.9 a	80.5 ± 1.4 a	27.3 ± 1.3 a	9.9 ± 0.7 a
W3K0	55.1 ± 0.9 b	70.1 ± 0.5 b	83.2 ± 0.4 a	28.1 ± 1.3 a	10.1 ± 1.1 a
W3K1	54.5 ± 0.5 b	69.7 ± 0.2 b	84.8 ± 0.2 b	30.3 ± 0.6 b	10.9 ± 0.5 a
W3K2	52.9 ± 0.4 a	66.9 ± 1.6 a	83.5 ± 0.6 a	30.6 ± 0.9 b	9.5 ± 0.4 a
Y1K0	60.5 ± 0.2 a	73.1 ± 0.5 a	86.3 ± 0.3 a	25.8 ± 0.5 a	10.9 ± 0.1 a
Y1K1	61.8 ± 0.8 a	74.0 ± 0.2 ab	86.0 ± 0.8 a	24.2 ± 0.8 a	10.3 ± 0.8 a
Y1K2	61.5 ± 0.5 a	74.6 ± 0.7 b	87.3 ± 0.9 a	25.7 ± 0.8 a	11.7 ± 0.9 a
Y2K0	55.6 ± 0.1 b	64.7 ± 0.1 c	86.1 ± 0.3 b	30.5 ± 0.3 b	10.9 ± 0.7 a
Y2K1	54.7 ± 0.7 ab	63.5 ± 0.9 b	84.1 ± 0.2 a	29.4 ± 0.7 ab	11.2 ± 0.9 a
Y2K2	54.4 ± 0.2 a	62.3 ± 0.4 a	83.3 ± 0.8 a	28.9 ± 0.7 a	9.9 ± 0.5 a
Y3K0	56.4 ± 0.6 a	70.5 ± 0.9 a	87.1 ± 0.1 a	30.7 ± 0.5 b	10.5 ± 0.1 a
Y3K1	57.4 ± 0.3 b	72.5 ± 0.4 b	85.2 ± 1.2 a	27.8 ± 1.1 a	10.0 ± 0.7 a
Y3K2	57.5 ± 0.1 b	71.7 ± 0.7 ab	85.3 ± 1.1 a	27.7 ± 1.1 a	10.5 ± 0.4 a
P1K0	58.8 ± 0.3 a	79.8 ± 0.3 c	86.2 ± 0.5 b	27.4 ± 0.2 b	11.6 ± 0.3 a
P1K1	58.0 ± 0.1 a	72.4 ± 0.3 a	83.2 ± 0.5 a	25.2 ± 0.6 a	11.2 ± 0.7 a
P1K2	63.5 ± 1.2 b	77.3 ± 0.2 b	87.3 ± 0.4 b	23.8 ± 1.6 a	11.9 ± 1.8 a
P2K0	64.1 ± 0.7 b	78.9 ± 0.2 b	87.2 ± 0.4 a	23.1 ± 0.8 a	10.9 ± 0.6 a
P2K1	66.0 ± 0.2 b	79.0 ± 0.1 b	87.0 ± 0.5 a	21.0 ± 0.4 a	10.8 ± 0.1 a
P2K2	59.8 ± 2.8 a	77.1 ± 0.3 a	89.6 ± 0.5 b	29.8 ± 3.0 b	14.2 ± 0.5 b
P3K0	56.1 ± 0.2 ab	70.0 ± 0.8 a	84.3 ± 0.5 a	28.2 ± 0.4 a	9.5 ± 0.5 a
P3K1	56.3 ± 0.4 b	76.7 ± 0.4 b	86.0 ± 0.7 a	29.7 ± 0.7 a	10.2 ± 0.4 a
P3K2	55.4 ± 0.3 a	70.2 ± 0.5 a	84.4 ± 1.2 a	28.9 ± 1.5 a	10.1 ± 0.9 a

^a^ Data are means ± standard deviations (*n* = 3). Values in the same column and the same variety with different letters are significantly different (*p* < 0.05). ^b^ To, gelatinization onset temperature; Tp, gelatinization peak temperature; Tc, gelatinization conclusion temperature; ΔT, gelatinization temperature range (Tc–To); ΔH, gelatinization enthalpy.

**Table 5:** Pasting parameters of starches ^a^.

	PV (mPa s) ^b^	HV (mPa s) ^b^	BV (mPa s) ^b^	FV (mPa s) ^b^	SV (mPa s) ^b^	P_time_ (min) ^b^	P_temp_ (°C) ^b^
W1K0	5959 ± 29 b	2927 ± 8 b	3032 ± 25 b	3557 ± 3 b	630 ± 7 a	4.80 ± 0.07 ab	74.2 ± 0.1 a
W1K1	6104 ± 48 c	2929 ± 13 b	3175 ± 36 c	3602 ± 30 c	674 ± 18 a	4.69 ± 0.04 a	74.2 ± 0.0 a
W1K2	5570 ± 35 a	2841 ± 19 a	2729 ± 20 a	3515 ± 8 a	674 ± 27 a	4.91 ± 0.08 b	75.0 ± 0.0 b
W2K0	5586 ± 16 a	2456 ± 37 a	3130 ± 32 a	3253 ± 13 a	797 ± 50 a	4.71 ± 0.04 b	73.3 ± 0.1 b
W2K1	5523 ± 8 a	2460 ± 23 a	3063 ± 15 a	3253 ± 28 a	793 ± 16 a	4.82 ± 0.04 c	73.1 ± 0.6 b
W2K2	6073 ± 53 b	2421 ± 23 a	3652 ± 39 b	3219 ± 47 a	798 ± 26 a	4.33 ± 0.00 a	70.1 ± 0.0 a
W3K0	5424 ± 20 a	2202 ± 38 a	3222 ± 19 b	3101 ± 6 a	899 ± 33 a	4.60 ± 0.00 b	72.5 ± 0.0 b
W3K1	5173 ± 31 a	2288 ± 54 a	2885 ± 30 a	3138 ± 29 a	850 ± 25 a	4.76 ± 0.04 c	73.0 ± 0.5 b
W3K2	6261 ± 52 b	2623 ± 25 b	3638 ± 62 c	3429 ± 34 b	806 ± 54 a	4.53 ± 0.00 a	69.7 ± 0.4 a
Y1K0	5347 ± 10 a	2730 ± 13 a	2617 ± 6 a	3568 ± 36 a	838 ± 22 a	4.82 ± 0.04 a	75.3 ± 0.5 a
Y1K1	5297 ± 45 a	2722 ± 29 a	2575 ± 55 a	3557 ± 38 a	835 ± 58 a	4.78 ± 0.04 a	75.2 ± 0.6 a
Y1K2	5362 ± 9 a	2822 ± 16 b	2540 ± 10 a	3641 ± 37 a	819 ± 47 a	5.00 ± 0.00 b	75.6 ± 0.4 a
Y2K0	5525 ± 9 a	2474 ± 44 a	3050 ± 42 a	3316 ± 28 a	842 ± 26 a	4.71 ± 0.04 c	73.3 ± 0.1 c
Y2K1	6316 ± 7 b	2729 ± 33 c	3587 ± 31 b	3517 ± 55 b	788 ± 22 a	4.53 ± 0.00 b	72.2 ± 0.4 b
Y2K2	6305 ± 10 b	2642 ± 10 b	3663 ± 11 c	3446 ± 82 ab	804 ± 77 a	4.42 ± 0.04 a	71.1 ± 0.5 a
Y3K0	5661 ± 29 b	2865 ± 7 b	2796 ± 35 a	3699 ± 42 b	834 ± 49 a	4.76 ± 0.04 a	74.5 ± 0.5 a
Y3K1	5435 ± 41 a	2623 ± 22 a	2812 ± 40 a	3453 ± 38 a	830 ± 18 a	4.76 ± 0.04 a	74.5 ± 0.2 a
Y3K2	5663 ± 21 b	2828 ± 19 b	2836 ± 23 a	3677 ± 32 b	849 ± 40 a	4.82 ± 0.04 a	74.2 ± 0.1 a
P1K0	5326 ± 41 a	2639 ± 27 a	2686 ± 60 a	3327 ± 14 a	687 ± 24 a	5.07 ± 0.00 c	76.1 ± 0.5 b
P1K1	5960 ± 47 b	2638 ± 41 a	3322 ± 8 b	3351 ± 48 a	713 ± 11 a	4.62 ± 0.04 a	75.0 ± 0.0 a
P1K2	5283 ± 49 a	2563 ± 46 a	2721 ± 11 a	3290 ± 16 a	727 ± 30 a	4.93 ± 0.07 b	78.1 ± 0.0 c
P2K0	4773 ± 13 a	2557 ± 11 a	2216 ± 7 a	3293 ± 24 a	736 ± 14 a	5.09 ± 0.04 b	78.4 ± 0.4 a
P2K1	4794 ± 44 a	2537 ± 24 a	2257 ± 25 a	3342 ± 15 a	805 ± 38 a	4.98 ± 0.04 a	78.7 ± 0.4 a
P2K2	4900 ± 88 a	2556 ± 55 a	2343 ± 37 b	3358 ± 90 a	802 ± 36 a	4.98 ± 0.04 a	78.4 ± 0.5 a
P3K0	5922 ± 29 c	2889 ± 76 b	3033 ± 48 b	3690 ± 59 b	801 ± 33 a	4.64 ± 0.04 a	74.1 ± 0.1 b
P3K1	5405 ± 72 a	2650 ± 26 a	2755 ± 66 a	3558 ± 49 a	908 ± 49 b	4.82 ± 0.04 b	75.0 ± 0.0 c
P3K2	5767 ± 39 b	2683 ± 4 a	3084 ± 36 b	3580 ± 40 ab	897 ± 43 b	4.60 ± 0.00 a	73.4 ± 0.1 a

^a^ Data are means ± standard deviations (*n* = 3). Values in the same column and the same variety with different letters are significantly different (*p* < 0.05). ^b^ PV, peak viscosity; HV, hot viscosity; BV, breakdown viscosity (PV–HV); FV, final viscosity; SV, setback viscosity (FV–HV); P_time_, peak time; P_temp_, pasting temperature.

## Data Availability

The original contributions presented in the study are included in the article/Supplementary Materials, further inquiries can be directed to the corresponding authors.

## Supplementary Materials

The following supporting information can be downloaded at https://www.mdpi.com/article/10.3390/foods13233890/s1: Figure S1: Temperature and rainfall information during the growth stage of sweet potato. HT and LT indicate the highest and lowest temperatures in a month, respectively.
