# Supplementary material for: Influence of Elevated Potassium Fertilization on Structural and Functional Properties of Sweet Potato Root Tuber Starch
Source: Foods. 2024 Dec 2;13(23):3890. doi: 10.3390/foods13233890 (PMC11640388; doi:10.3390/foods13233890)
Supplement: Supplementary file 1 [file foods-13-03890-s001.zip › foods-3292398-supplementary.pdf]

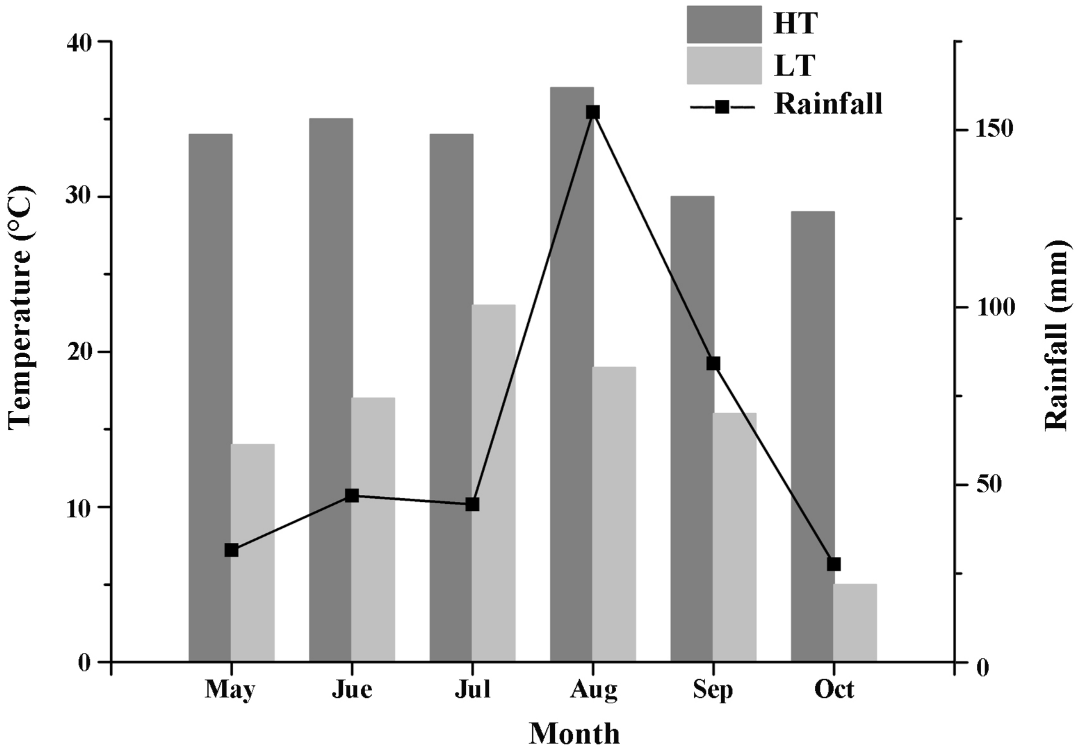

Figure S1 Temperature and rainfall information during the growth stage of sweetpotato. HT and LT indicate the highest and lowest temperatures in a month, respectively
